# Proteomic profiling for the identification of serum diagnostic biomarkers for abdominal and thoracic aortic aneurysms

**DOI:** 10.1186/1477-5956-11-27

**Published:** 2013-06-27

**Authors:** Kazumi Satoh, Tomoko Maniwa, Teiji Oda, Ken-ichi Matsumoto

**Affiliations:** 1 Department of Biosignaling and Radioisotope Experiment, Interdisciplinary Center for Science Research, Organization for Research, Shimane University, Enya-cho, Izumo 693-8501, Japan; 2 Division of Cardiovascular and Thoracic Surgery, Department of Surgery, Faculty of Medicine, Shimane University, Izumo, Japan

**Keywords:** Abdominal Aortic Aneurysms, Differential Level Protein, iTRAQ, Serum Proteomics, Thoracic Aortic Aneurysms

## Abstract

**Background:**

Aortic aneurysm is an increasingly common vascular disorder with fatal implication. However, there is no established diagnosis other than that based on aneurysmal size. For this purpose, serum protein biomarkers for aortic aneurysms are valuable. Although most of the studies on serum biomarker discovery have been based on comparison of serum proteins from the patient group with those from the healthy group, we considered that comparison of serial protein profiles such as those in presurgical and postsurgical sera within one patient would facilitate identification of biomarkers since the variability of serial protein profiles within one patient is smaller than that between groups. In this study, we examined serum proteins with differential levels in postsurgery compared with those in presurgery after the removal of aneurysmal tissues in abdominal aortic aneurysm (AAA) and thoracic aortic aneurysm (TAA) patients in order to identify potential serum biomarkers for AAAs and TAAs.

**Results:**

A proteomic approach with an isobaric tag for relative and absolute quantitation (iTRAQ) labeling followed by nano liquid chromatography (nanoLC)-matrix-assisted laser desorption ionization (MALDI)-time of flight (TOF/TOF)-tandem mass spectrometry (MS/MS) was used. In the sera of patients with AAAs and TAAs, a total of 63 and 71 proteins with differential levels were further narrowed down to 6 and 8 increased proteins (≧1.3 fold, postsurgical *vs*. presurgical) (*p* < 0.05, patient *vs*. control) and 12 and 17 decreased proteins (< 0.77 fold, postsurgical *vs*. presurgical) (*p* < 0.05, patient *vs*. control) in postsurgical sera compared with those in presurgical sera, respectively. All of the increased proteins in postsurgical sera of both AAA and TAA patients included several known acute-phase proteins. On the other hand, in the decreased proteins, we found intriguing molecules such as α-2-macroglobulin, gelsolin, kallistatin, and so on. Among them, we confirmed that kallistatin in both AAA and TAA patients and α-2-macroglobulin in TAA patients showed decrease levels in postsurgical sera similar to those in control sera by Western blot analysis with other sera from AAA and TAA patients.

**Conclusions:**

Taken together, our findings suggest that Kallistatin and α-2-macroglobulin are potential serum biomarkers for both AAA and TAA and TAA, respectively.

## Background

Aortic aneurysm is a severe cardiovascular disease with high mortality and morbidity. It is a complex disease with genetic and environmental risk factors. Aneurysms are described as abdominal aortic aneurysms (AAAs) and thoracic aortic aneurysms (TAAs) in terms of their anatomic location. It is well known that both AAAs and TAAs are associated with male gender, advanced age, cigarette smoking, hypertension, and chronic obstructive pulmonary disease. Both thoracic and abdominal aortas are large vascular conduits with similarities in their cellular components. However, thoracic and abdominal aortas are distinct in their biochemical and biomechanical properties, proteolytic profiles, inflammatory response, and genetic factors [1]. These differences are implicated in the distinct onset and expansion of aortic aneurysms in the thoracic and abdominal regions. A significant difference occurs in the distribution of aneurysms, and the most common location for aneurysms is in the infrarenal abdominal aorta, followed by the ascending thoracic aorta. In addition, it has been shown that intimal atherosclerosis, chronic transmural inflammation and elastic media destruction are associated with AAA growth, but many of TAAs arise in the absence of atherosclerotic plaque deposition. Preferably, TAAs are associated with the medial degeneration that is commonly related to the loss of vascular smooth muscle cells and destruction of medial elastic fibers [2].

Most patients with aortic aneurysms are asymptomatic until rupture of the aneurysm, which is often fatal. This disease is diagnosed when the diameter reaches 30 mm or more. However, there is no established medical therapeutics for small aneurysms. For this purpose, plasma/serum protein biomarkers are available. Plasma or serum is considered a suitable body fluid for biomarker discovery since it is readily obtained via the least invasive mode without the risk of surgery. Studies are needed to find the serum/plasma biomarkers other than those for diagnosis based on aneurysmal size for the prediction of aneurysmal risk [3]. Proteome profiling of serum could also facilitate the discovery of AAA and/or TAA biomarkers for prognosis and therapeutic purposes for patients with aortic aneurysms. Circulating markers for the progression of AAA have been investigated [4,5]. These include extracellular matrix, matrix modulating proteases and their inhibitors, thrombogenic proteins, cytokines and inflammatory proteins, and lipids. Although a few of them have clinical potential for diagnosis, further confirmative and larger studies are needed. On the other hand, there were no reports about circulating biomarkers for TAA so far. It is conceivable that pathological disparities between AAA and TAA would bear different serum/plasma biomarkers related to each aneurysm.

The wide dynamic range of proteins and the presence of a few major proteins such as albumin and immunoglobulin complicate the analysis of serum/plasma proteomes. In addition, serum/plasma biomarkers would be factors released in very small amounts into the blood stream, making it difficult to detect them among other serum/plasma proteins that exist in much larger amounts. The removal of major proteins is a prerequisite for the identification of serum/plasma biomarkers in small amounts. This would increase the sample loading capacity and improve the efficiency of detection of proteins that exist in small amounts.

On the other hand, most of the studies on plasma/serum biomarker discovery have been based on comparison of the mean values of plasma/serum proteins from the patient group with those from the healthy volunteer group. However, this approach sometimes gives rise to a large variation within each group, leading to masking of the variation that occurs between groups. Therefore, it is expected that the variability of serial protein profiles within one individual is smaller than that between groups [6]. Comparison of serial protein profiles within one individual would facilitate identification of biomarkers. We also expected that the postsurgical level of a certain protein would be restored to a level similar to that in normal control sera after resection of lesion parts. We anticipated that monitoring changes in serum proteomes that occur within each patient after surgical resection of aortic aneurysm tissues would readily provide biomarkers for clinical diagnosis and treatment and for understanding the molecular mechanisms underlying the development of AAA and TAA.

Recently, quantitative proteome analyses using tandem mass spectrometry (MS) with an iTRAQ labeling strategy have been developed and successfully applied to biomarker discovery for many conditions in both tissue [7] and serum samples [8]. In this study, serum proteins with differential levels in postsurgery compared with those in presurgery for the removal of aneurysmal tissues in AAA and TAA patients were investigated with iTRAQ labeling followed by nanoLC-MALDI-TOF/TOF-MS/MS.

## Results

### Proteomic analyses of serum proteins with differential levels in postsurgical sera compared with those in presurgical sera of AAA and TAA patients

**Figure 1:**
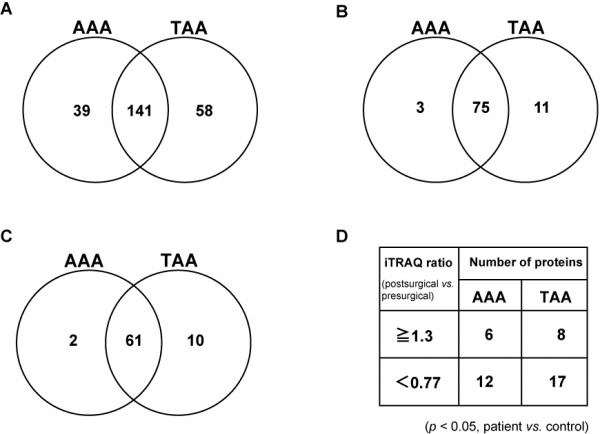
**Venn diagrams showing the number of proteins with differential levels in postsurgical sera compared with those in presurgical sera of patients with AAA and TAA.** (**A**) Number of proteins that were identified in at least one sample among 7 samples in each group. (**B**) Number of proteins that were identified in at least 6 samples among 7 samples in each group. (**C**) Number of remaining proteins after exclusion of proteins for which iTRAQ ratios at the second time point (T2) to those at the first time point (T1) showed ≧ 1.3 fold or < 0.77 fold in at least two samples in 4 control sera (T2 *vs.* T1). (**D**) Numbers of proteins with significantly increased (≧ 1.3 fold, postsurgical *vs.* presurgical) and decreased level ratios (< 0.77 fold, postsurgical *vs.* presurgical) that were identified in at least 6 samples among 7 samples in sera of AAA and TAA patients and with statistical significant levels of iTRAQ ratio of postsurgical/presugical of patients’ sera compared with that of T2/T1 volunteers’ sera (*p* < 0.05, patient *vs.* control).

Presurgery and postsurgery serum samples were obtained from 7 AAA and 7 TAA patients who underwent aortic aneurysm resection. Protein levels in postsurgical sera were compared with those in presurgical sera using iTRAQ labeling coupled to nanoLC-MALDI-TOF/TOF-MS/MS followed by ProteinPilot analysis. The average iTRAQ ratios of peptides in postsurgical sera to those in presurgical sera were calculated. A total of 180 differential level proteins in 7 AAA patients’ sera and 199 proteins in 7 TAA patients’ sera with 141 proteins detected in common in both groups were identified in at least one patient’s serum (Figure 1A). Among those proteins, 78 differential level proteins in 7 AAA patients’ sera and 86 proteins in 7 TAA patients’ sera with 75 proteins detected in both groups were identified in at least 6 patients’ sera in each group (Figure 1B). Relative quantitation by ProteinPilot analysis is based on statistical analysis. However, since most biochemical methods tend to have technical variation, we considered an additional cutoff value at 1.3-fold change (≧1.3 fold or < 0.77 fold, postsurgical *vs*. presurgical) for iTRAQ ratios for the selection of serum proteins with differential levels in postsurgical sera compared with those in presurgical sera [9-11]. Furthermore, among them, proteins in at least two samples in 4 volunteers’ normal control sera for which iTRAQ ratios at the second time point (T2) to those at the first time point (T1) showed ≧1.3 fold or < 0.77 fold (T2 *vs*. T1) were excluded. We used the “unused ProtScore” value (> 2) in ProteinPilot for an index of protein confidence (> 99% confidence). Consequently, 63 proteins with differential levels in 7 AAA patients’ sera and 71 proteins in 7 TAA patients’ sera with 61 proteins detected in both groups were identified in postsurgical sera compared with those in presurgical sera with highly stringent criteria for protein identification (Figure 1C). Furthermore, the proteins that show statistical significant levels of postsurgical/presugical (patients’ sera) compared with those of T2/T1 (control sera) (*p* < 0.05, patient *vs*. control) were selected. Finally, 6 and 8 proteins with significantly increased levels (≧1.3 fold, postsurgical *vs*. presurgical) and 12 and 17 proteins with significantly decreased levels (< 0.77 fold, postsurgical *vs*. presurgical) were identified in AAA and TAA patients’ sera, respectively (Figure 1D).

Increased (≧1.3 fold, postsurgical *vs*. presurgical) and decreased (< 0.77 fold, postsurgical *vs*. presurgical) serum proteins in postsurgical sera compared with those in presurgical sera of AAA patients (Table 1) and TAA patients (Table 2) are listed in the order of iTRAQ ratios, respectively. Among them, we investigated the correlation between the ratios of proteins in postsurgical sera to those in presurgical sera with gender difference of patients that provided blood samples (Additional file 1). In increased proteins in postsurgical sera of AAA patients (Table 1), leucine-rich α-2-glycoprotein (LRG1) was detected [male group (AAA patients #AAA1, #AAA2, #AAA3, #AAA4 and #AAA5) *vs*. female group (#AAA6 and #AAA7), *p* = 0.0198 (unpaired *t*-test)]. In increased proteins in postsurgical sera of TAA patients (Table 2), serum amyloid A protein was significantly detected [male group (TAA patients #TAA1, #TAA4, #TAA5, and #TAA6) *vs*. female group (#TAA2, #TAA3 and #TAA7), *p* = 0.0046]. In decreased proteins in postsurgical sera of TAA patients (Table 2), gelsolin (male *vs*. female, *p* = 0.0357), histidine-rich glycoprotein (male *vs*. female, *p* = 0.0132), fibronectin (male *vs*. female, *p* = 0.0101), α-2-macroglobulin (male *vs*. female, *p* = 0.0198), and inter-α-trypsin inhibitor heavy chain H2 (male *vs*. female, *p* = 0.0170) were significantly detected. Furthermore we also examined the correlation between the ratios of proteins in postsurgical sera to those in presurgical sera with the number of days of blood sampling before and after surgery (Additional file 1). In increased proteins in postsurgical sera of AAA patients (Table 1), serum amyloid A protein was significantly detected [grouping based on the number of days before surgery, less than 1 week group (#AAA1, #AAA3 and #AAA6) *vs*. more than 1 week group (#AAA2, #AAA4, #AAA5 and #AAA7), *p* = 0.0374].

**Table 1:** Proteins with differential levels in postsurgical sera compared with those in presurgical sera of AAA patients

**Unused protScore**^**a**^	**% Coverage**^**b**^	**Peptides**^**c **^**(95%)**	**Uniplot number**	**Gene symbol**	**Protein name**	**iTRAQ ratio**^**d **^**average ± SE**	***p *value**^**e**^	**Molecular function**
						**(postsurgical *vs*. presurgical)**	**(patient *vs*. control)**	
***Increased proteins***								
37.7	86.9	28	P02735	SAA1	Serum amyloid A protein	8.09 ± 3.60	-^f^	Acute phase reactant
48.8	56.5	37	P01011	SERPINA3	Alpha-1-antichymotrypsin	1.88 ± 0.12	0.0006	Protease inhibitor
53.1	67.7	56	P02763	ORM1	Alpha-1-acid glycoprotein 1	1.85 ± 0.21	0.0250	Transporter
26.2	54.5	14	P02750	LRG1	Leucine-rich alpha-2-glycoprotein	1.85 ± 0.15	0.0032	Unknown
26.5	40.1	13	P02748	C9	Complement component C9	1.36 ± 0.09	0.0135	Complement
116.9	80.9	139	P01009	SERPINA1	Alpha-1-antitrypsin	1.32 ± 0.06	0.0081	Protease inhibitor
***Decreased proteins***								
26.1	30.4	12	P06396	GSN	Gelsolin	0.55 ± 0.03	0.0000	Actin scavenger
37.3	54.2	29	P02765	AHSG	Alpha-2-HS-glycoprotein	0.61 ± 0.05	0.0002	Extracellular matrix
18.5	74.0	17	P02652	APOA2	Apolipoprotein A-II	0.64 ± 0.05	0.0214	Transporter
14.1	60.7	9	P02753	RBP4	Retinol-binding protein 4	0.65 ± 0.05	0.0005	Transfer/carrier protein
20.9	36.3	10	P29622	SERPINA4	Kallistatin	0.68 ± 0.06	0.0196	Protease inhibitor
10.0	25.1	6	P27169	PON1	Serum paraoxonase/arylesterase 1	0.69 ± 0.06	0.0010	Hydrolase
89.6	89.9	110	P02647	APOA1	Apolipoprotein A-I	0.70 ± 0.04	0.0238	Transporter
149.6	49.4	90	P02751	FN1	Fibronectin	0.72 ± 0.06	0.0330	Extracellular matrix
163.9	82.5	176	P02787	TF	Serotransferrin	0.73 ± 0.04	0.0004	Transfer/carrier protein
36.9	50.3	20	P04196	HRG	Histidine-rich glycoprotein	0.73 ± 0.03	0.0005	Adapter protein
234.7	81.7	207	P01023	A2M	Alpha-2-macroglobulin	0.75 ± 0.04	0.0036	Signaling molecule
44.3	35.7	26	P19823	ITIH2	Inter-alpha-trypsin inhibitor heavy chain H2	0.77 ± 0.06	0.0024	Protease inhibitor

^a^A score of protein confidence(ProtScore) for a detected protein that is calculated from the peptide confidence from spectra that are not already “used” by higher scoring proteins in the experiments.

^b^The percentage of matching amino acids from identified peptides.

^c^The number of distinct peptides with at least 95% confidence in the experiments.

^d^iTRAQ ratio of postsurgical sera compared with those in presurgical sera (postsurgical *vs.* presurgical).

^e^Statistical analysis of iTRAQ ratio of postsurgery/presurgery of patients’ sera compared with that of T2/T1 of control volunteers’ sera (patient *vs.* control).

^f^Protein that did not appear in control sera.

**Table 2:** Proteins with differential levels in postsurgical sera compared with those in presurgical sera of TAA patients

**Unused protScore**^**a**^	**% Coverage**^**b**^	**Peptides**^**c **^**(95%)**	**Uniplot number**	**Gene symbol**	**Protein name**	**iTRAQ ratio**^**d **^**average ± SE**	***p *value**^**e**^	**Molecular function**
						**(postsurgical *vs*.presurgical)**	**(patient *vs*. control)**	
***Increased proteins***								
46.2	82.0	48	P02735	SAA1	Serum amyloid A protein	15.65 ± 3.40	-^f^	Acute phase protein
16.0	34.8	9	P02741	CRP	C-reactive protein	4.31 ± 0.54	-^f^	Acute phase protein
60.0	73.6	68	P02763	ORM1	Alpha-1-acid glycoprotein 1	2.61 ± 0.18	0.0002	Transporter
62.0	68.3	47	P01011	SERPINA3	Alpha-1-antichymotrypsin	2.39 ± 0.21	0.0010	Protease inhibitor
29.8	59.1	24	P02750	LRG1	Leucine-rich alpha-2-glycoprotein	2.11 ± 0.30	0.0235	Unknown
123.8	80.6	151	P01009	SERPINA1	Alpha-1-antitrypsin	1.50 ± 0.15	0.0397	Protease inhibitor
28.3	32.7	16	P02748	C9	Complement component C9	1.43 ± 0.15	0.0469	Complement
16.5	21.7	8	P09871	C1S	Complement C1s subcomponent	1.30 ± 0.07	-^f^	Complement
***Decreased proteins***								
30.2	38.0	15	P06396	GSN	Gelsolin	0.49 ± 0.05	0.0002	Actin scavenger
38.9	54.5	30	P02765	AHSG	Alpha-2-HS-glycoprotein	0.51 ± 0.09	0.0008	Extracellular matrix
19.3	89.0	14	P02652	APOA2	Apolipoprotein A-II	0.52 ± 0.09	0.0258	Transporter
14.2	27.2	7	P29622	SERPINA4	Kallistatin	0.56 ± 0.08	0.0085	Protease inhibitor
95.4	92.1	97	P02647	APOA1	Apolipoprotein A-I	0.59 ± 0.09	0.0473	Transporter
16.5	53.7	10	P02753	RBP4	Retinol-binding protein 4	0.60 ± 0.10	0.0027	Transfer/carrier protein
183.4	83.7	171	P02787	TF	Serotransferrin	0.60 ± 0.08	0.0021	Transfer/carrier protein
23.1	67.6	11	O95445	APOM	Apolipoprotein M	0.61 ± 0.05	0.0050	Transporter
36.3	46.3	19	P04196	HRG	Histidine-rich glycoprotein	0.61 ± 0.07	0.0061	Adapter protein
156.2	49.9	98	P02751	FN1	Fibronectin	0.65 ± 0.07	0.0261	Extracellular matrix
13.3	27.3	7	P27169	PON1	Serum paraoxonase/arylesterase 1	0.66 ± 0.07	0.0011	Hydrolase
16.4	22.3	7	P03952	KLKB1	Plasma kallikrein	0.66 ± 0.09	0.0191	Protease
12.0	78.2	7	P02655	APOC2	Apolipoprotein C-II	0.66 ± 0.14	0.0387	Transporter
289.1	78.8	228	P01023	A2M	Alpha-2-macroglobulin	0.66 ± 0.05	0.0027	Signaling molecule
59.1	41.9	34	P19823	ITIH2	Inter-alpha-trypsin inhibitor heavy chain H2	0.68 ± 0.08	0.0043	Protease inhibitor
28.4	38.4	14	P43652	AFM	Afamin	0.70 ± 0.08	0.0041	Transfer/carrier protein
30.5	44.7	18	P05546	SERPIND1	Heparin cofactor 2	0.76 ± 0.09	0.0424	Protease inhibitor

^a^A score of protein confidence(ProtScore) for a detected protein that is calculated from the peptide confidence from spectra that are not already “used” by higher scoring proteins in the experiments.

^b^The percentage of matching amino acids from identified peptides.

^c^The number of distinct peptides with at least 95% confidence in the experiments.

^d^iTRAQ ratio of postsurgical sera compared with those in presurgical sera (postsurgical *vs.* presurgical).

^e^Statistical analysis of iTRAQ ratio of postsurgery/presurgery of patients’ sera compared with that of T2/T1 of control volunteers’ sera (patient *vs.* control).

^f^Protein that did not appear in control sera.

As mentioned below, among them two proteins (gelsolin and kallistatin) in patients’ sera with AAA and three proteins (gelsolin, kallistatin and afamin) in patients’ sera with TAA were finally examined by Western blot analyses with other presurgery and postsurgery blood samples. Furthermore, a positive acute-phase protein, α-2-macroglobulin was also confirmed by Western blot analysis.

### Verification of iTRAQ ratio by Western blot analysis

**Figure 2:**
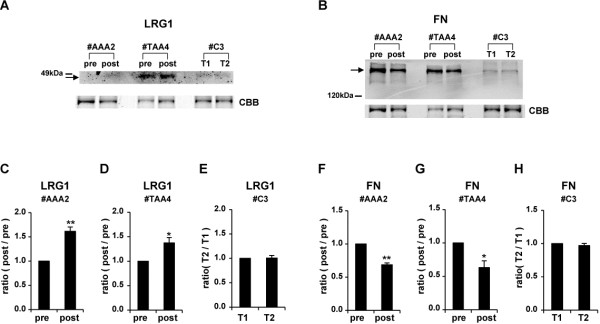
**Confirmation of iTRAQ ratio by Western blot analyses.** Western blot analyses with anti-leucine-rich α-2-glycoprotein (referred to as LRG1) (**A**) and anti-fibronectin (FN) (**B**) antibodies in presurgical (pre) and postsurgical (post) sera of patient #AAA2 and patient #TAA4 were performed. Representative photos are shown. To confirm equal levels of proteins per lane, non-specific proteins stained with Coomassie Brilliant Blue (CBB) are shown in the lowest panel. The intensity of each band that reacted with the corresponding antibody indicated by an arrow was measured. Ratios of levels of LRG1 (**C**) and fibronectin (**F**) in postsurgical sera compared with those in presurgical sera (1.0) in #AAA2 were determined by band intensity. Ratios of levels of LRG1 (**D**) and fibronectin (**G**) in postsurgical sera compared with those in presurgical sera (1.0) in #TAA4 were also determined by band intensity. Furthermore, Western blot analyses with anti-LRG1 (**A**) and anti-FN (**B**) at two different time points (T1 and T2) in serum of a control volunteer #C3 were performed. Ratios of levels of LRG1 (**E**) and fibronectin (**H**) at T2 compared with those at T1 (1.0) in serum of the control volunteer were determined by band intensity. Means ± SE of quadruplicate experiments were calculated, and statistical analysis was performed using the paired *t*-test. ** *p* < 0.01. *p < 0.05.

To confirm the accuracy of the quantitative results of iTRAQ ratios, some proteins were quantified again by Western blot analysis. The relative amounts of LRG1 (iTRAQ ratio = 1.99, postsurgical *vs*. presurgical) and fibronectin (iTRAQ ratio = 0.69, postsurgical *vs*. presurgical) for AAA patient #AAA2 (Figures 2A,B,C,F) and LRG1 (iTRAQ ratio = 2.24, postsurgical *vs*. presurgical) and fibronectin (iTRAQ ratio = 0.63, postsurgical *vs*. presurgical) for TAA patient #TAA4 (Figures 2A,B,D,G) in postsurgical sera compared with those in presurgical sera were examined by Western blot analysis. The ratios of levels of LRG1 and fibronectin in postsurgical sera compared with those in presurgical sera (1.0) in the AAA and TAA patients were determined by band intensity, and they were 1.62-fold (Figure 2C) and 0.69-fold (Figure 2F), respectively, in sera of AAA patients and 1.37-fold (Figure 2D) and 0.63-fold (Figure 2G), respectively, in sera of TAA patients. Almost the same results were obtained in AAA patient #AAA3 and TAA patient #TAA1 (data not shown). Similarly, iTRAQ ratios of LRG1 (0.93) and fibronectin (0.96) in serum of a control volunteer #C3 collected at two different time points (T1 and T2) were almost identical to the ratios determined by Western blot analysis (LRG1, 1.01; fibronectin, 0.97) (Figures 2A,B,E,H). These results indicated that iTRAQ ratios are almost consistent with quantitative results obtained by Western blot analysis.

### Confirmation of differential levels of α-2-macroglobulin and kallistatin in presurgical and postsurgical sera by Western blot analysis

All of the proteins on the list of serum proteins that were increased (≧ 1.3 fold, postsurgical *vs*. presurgical) in postsurgical sera compared with those in presurgical sera of AAA patients and TAA patients (Tables 1 and 2) were inflammatory response markers with increased levels during acute-phase response (see Discussion) [12-16]. These proteins include serum amyloid A protein, α-1-antichymotrypsin, α-1-acid glycoprotein 1, LRG1, complement component C9, and α-1-antitrypsin in both AAA and TAA patients (Tables 1 and 2). C-reactive protein and complement C1s subcomponent were also increased in postsurgical sera of TAA patients (Table 2).

Intriguingly, five negative acute-phase proteins (retinol-binding protein 4 [17], fibronectin [18], serotransferrin [19], histidine-rich glycoprotein [20] and inter-α-trypsin inhibitor heavy chain H2 [21]) were decreased in postsurgical sera compared with those in presurgical sera of both AAA and TAA patients. Some constituents of high-density lipoprotein (HDL) that are suppressed during the acute-phase response [22], apolipoprotein (apo) A-I, apo A-II, and serum paraoxonase/arylesterase 1 in the case of sera from AAA patients and apo A-I, apo A-II, apo C-II, apo M, and serum paraoxonase/arylesterase 1 in the case of sera from TAA patients, were also included in the decreased proteins.

**Figure 3:**
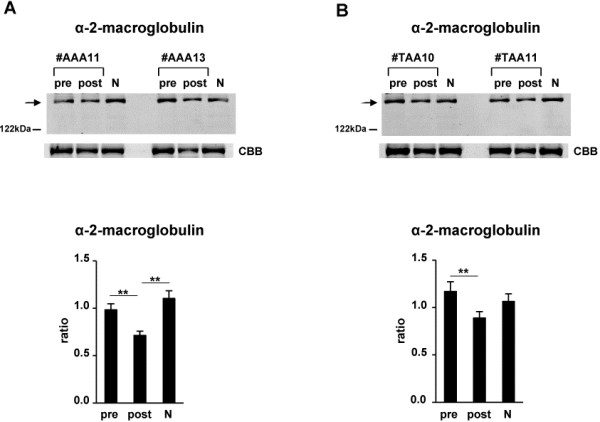
**Examination of the decreased level of α-2-macroglobulin in postsurgical sera compared with that in presurgical sera of both AAA and TAA patients, and restoration of its level to that in normal control sera.** Western blsot analyses with anti-α-2-macroglobulin antibody in presurgical (pre) and postsurgical (post) sera of 12 patients with AAA (**A**) and 10 patients with TAA (**B**) were performed as indicated by arrows. Representative photos of two each of patients (patient no. #AAA11, #AAA13, #TAA10 and #TAA11) as well as a mixture of 10 normal control sera (N) are shown. To confirm equal levels of proteins per lane, non-specific proteins stained with Coomassie Brilliant Blue are shown (CBB). The intensity of each band in presurgical and postsurgical sera of 12 AAA and 10 TAA patients and 10 normal control sera that reacted with anti-α-2-macroglobulin antibody was measured. Ratios of levels of α-2-macroglobulin in presurgical and postsurgical sera and normal control sera compared with that in the mixture of 10 normal control sera (1.0) were determined by band intensity. Means ± SE of triplicate experiments were calculated, and statistical analysis was performed using the paired *t*-test between presurgical and postsurgical data, whereas it was done using the unpaired *t*-test between postsurgical and normal control data. ** *p* < 0.01.

On the other hand, a positive acute-phase protein, α-2-macroglobulin, was unexpectedly decreased in postsurgical sera compared with that in presurgical sera of both AAA and TAA patients (Tables 1 and 2). Therefore, by Western blot analysis, we investigated the decreased levels of α-2-macroglobulin, using postsurgical sera compared with presurgical sera from 12 other AAA patients and 10 other TAA patients, and examined whether the level of α-2-macroglobulin in postsurgical sera is similar to that in normal control sera. If the level of α-2-macroglobulin in postsurgical sera returns to a level similar to that in control sera, α-2-macroglobulin would be a candidate of disease biomarkers for AAA and TAA. Consequently, we confirmed the decrease in α-2-macroglobulin in postsurgical sera (Figure 3). In addition, the level of α-2-macroglobulin in postsurgical sera of TAA patients (Figure 3B) but not in postsurgical sera of AAA patients (Figure 3A) was similar to that in normal control sera, indicating that α-2-macroglobulin would be a candidate of disease biomarkers for TAA.

**Figure 4:**
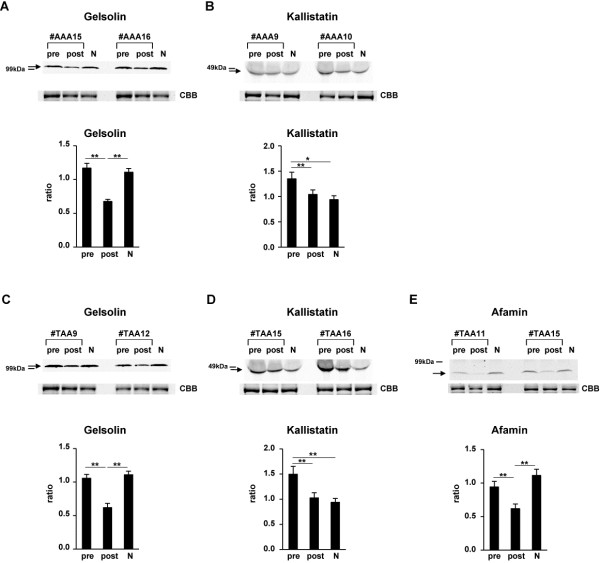
**Examination of the decreased levels of some proteins in postsurgical sera compared with those in presurgical sera of both AAA and TAA patients, and restoration of their levels to those in normal control sera.** Western blot analyses with anti-gelsolin (**A,C**), and anti-kallistatin (**B,D**), and anti-afamin (**E**) antibodies in presurgical (pre) and postsurgical (post) sera in 12 patients with AAA (**A,B**) and 10 patients with TAA (**C-E**) were performed as indicated by arrows. Representative photos of two each of patients concerning to each protein (patient no. #AAA15, #AAA16, #TAA9 and #TAA12 for gelsolin; #AAA9, #AAA10, #TAA15 and #TAA16 for kallistatin; #TAA11 and #TAA15 for afamin) as well as mixture of 10 normal control sera (N) are shown. To confirm equal levels of proteins per lane, non-specific proteins stained with Coomassie Brilliant Blue are shown (CBB). The intensity of each band in presurgical and postsurgical sera of 12 AAA and 10 TAA patients and 10 normal control sera that reacted with each antibody was measured. Ratios of levels of each protein in presurgical and postsurgical sera and normal control sera compared with that in the mixture of 10 normal control sera (1.0) were determined by band intensity. Means ± SE of triplicate experiments were calculated, and statistical analysis was performed using paired *t*-test between presurgical and postsurgical data, whereas it was done using unpaired *t*-test between presurgical or postsurgical and normal control data. ** *p* < 0.01. * *p* < 0.05.

Among other decreased serum proteins in postsurgical sera compared with those in presurgical sera, we were interested in proteins such as gelsolin and kallistatin in AAA patients and gelsolin, kallistatin and afamin in TAA patients, for which detailed analyses in connection with aortic aneurysms have not been performed. Therefore, we further investigated these decreased levels of proteins by Western blot analysis (Figure 4). As a result, we confirmed decreased levels of proteins such as gelsolin and kallistatin in postsurgical sera of AAA patients (Figures 4A,B) and gelsolin, kallistatin, and afamin in postsurgical sera of TAA patients (Figures 4C-E) compared with those in presurgical sera by Western blot analysis. Subsequently, we investigated whether the protein levels in postsurgical sera are similar to those in normal control sera. In consequence, only the levels of kallistatin in postsurgical sera of both AAA and TAA patients were similar to those in normal control sera (Figures 4B,D). The level of gelsolin in the postsurgical sera of AAA patients (Figure 4A) and the levels of gelsolin and afamin in postsurgical sera of TAA patients (Figures 4C,E) were not similar to those in normal control sera. Thus, in postsurgical sera compared to presurgical sera only kallistatin showed a decreased level similar to that in normal control sera, indicating that kallistatin would also be a candidate of disease biomarkers for AAA and TAA.

## Discussion

Serum proteins often possess information on diseases concerning the overall pathologic status of patients. Thus, circulating serum biomarkers could play an important role in the diagnosis of diseases and might also have a role in predicting progression of diseases. To identify critical molecular mechanisms underlying AAA formation, circulating serum biomarkers in patients and controls have been assessed in some case–control studies [4,5]. So far, most proteomic analyses attempting to reveal biological markers for AAA have been performed in AAA tissue samples [23]. Only one proteomic study for the identification of plasma biomarkers for AAA, which led to the identification of five differentially up-regulated proteins in plasma samples of AAA patients compared to control patients, has been published [24]. However, the broad range of molecular alterations that occur in postsurgical sera compared to presurgical sera of one patient with AAA or TAA has not been elucidated. We speculated that a comparison of presurgical and postsurgical protein profiles within one patient would facilitate discovery of disease biomarkers. We expected that if the postsurgical level of a certain protein that shows a differential level between presurgical and postsurgical sera is restored to a level similar to that in normal control sera after the resection of AAA or TAA, the protein would be a disease biomarker. In this study, using an approach with iTRAQ labeling followed by nanoLC-MALDI-TOF/TOF-MS/MS analysis, we determined proteomic profiles of proteins with differential levels in postsurgical sera compared with those in presurgical sera of AAA and TAA patients who underwent aortic aneurysm resections. Consequently, 6 and 8 proteins with increased level (≧1.3 fold, postsurgical *vs*. presurgical) and 12 and 17 proteins with decreased level (< 0.77 fold, postsurgical *vs*. presurgical) were identified in postsurgical sera compared with those in presurgical sera of AAA and TAA patients, respectively.

All of the proteins with increased levels (≧1.3 fold, postsurgical *vs*. presurgical) in postsurgical sera compared with those in presurgical sera of both AAA and TAA patients were positive acute-phase proteins such as serum amyloid A protein and α-1-acid glycoprotein 1. On the other hand, five serum proteins including retinol-binding protein 4, serotransferrin and others, and apolipoproteins such as apo A-I in HDL known as negative acute-phase proteins were decreased in postsurgical sera compared with those in presurgical sera of both AAA and TAA patients. Since postsurgical blood sampling was performed 4 to 20 days (Additional file 1) after surgical injury for aortic aneurysm resections, the increased levels of positive acute-phase proteins and the decreased levels of negative acute-phase proteins in postsurgical sera compared with those in presurgical sera might have been caused by the inflammatory response which is part of the normal response to surgical injury and not by specific resection of the diseased aortic aneurysmal tissues.

On the other hand, among other decreased proteins in postsurgical sera compared with those in presurgical sera, which we focused on and confirmed the decrease by Western blot analyses, we found that the decreased levels of kallistatin in both AAA and TAA patients’ postsurgical sera and α-2-macroglobulin in TAA patients’ postsurgical sera are restored to levels similar to those in normal control sera. These results indicate that kallistatin and α-2-macroglobulin would be candidates of disease biomarkers for both AAA and TAA patients and TAA patients, respectively.

Kallistatin was initially discovered as a tissue kallikrein inhibitor and was shown to have multifaceted effects such as anti-inflammation, anti-oxidative stress, and anti-angiogensis effects [25]. Administration of kallistatin prevented cardiac injury such as fibrosis and hypertrophy by inhibiting oxidative stress [26]. Since inflammation and oxidative-stress considerably arise in aortic aneurysmal tissues, the increase in kallistatin in patients’ sera would be necessary to prevent cardiac remodeling and oxidative cardiac damage.

The primary function of α-2-macroglobulin, one of the positive acute-phase proteins, is inhibition of a broad spectrum of proteases during tissue injury at sites of inflammation [27]. It has been reported that α-2-macroglobulin is a valuable serum marker for the diagnosis of cardiac diseases [28]. Aortic aneurysms are characterized by inflammation with infiltration of inflammatory cells, induction of inflammatory cytokines, and release of reactive oxygen species. However, the differences in the physical structures of abdominal and thoracic aortas might result in differences in the inflammatory responses of AAA and TAA [2]. It is known that a Th2-predominant immune response plays an important role in AAA, whereas a Th1-predominant immune response occurs in TAA. These differences in inflammatory responses in AAA and TAA may contribute to the fact that α-2-macroglobulin would be a candidate of disease biomarkers only in TAA patients.

Acosta-Martin et al. [24] identified 19 plasma diagnostic biomarker proteins potentially involved in AAA biological mechanisms using quantitative MS analysis. Among them, five proteins such as kallistatin, protein AMBP, gelsolin, heparin cofactor 2, α-2-antiplasmin were found in common in the 63 proteins disclosed in AAA patients’ sera in the present study. Moreover, they verified the increased levels of kallistatin and protein AMBP in individual plasma of AAA patients by Western blot analysis. In agreement with this, in the present study the level of kallistatin in presurgical sera was higher than in postsurgical sera and control sera as shown in Table 1 and Figure 4B. Furthermore, in the present study the level of protein AMBP in presurgical sera was moderately higher than that in postsurgical sera (iTRAQ ratio =1.17, presurgical *vs*. postsurgical). These evidences more strengthen the idea that kallistatin is a candidate of disease biomarkers for AAA.

So far some extracellular matrices such as aminoterminal propeptide of type III procollagen (PIIINP) and carboxyterminal propeptide of type I procollagen (PICP), thrombus-associated proteins such as fibrinogen, D-dimer and tissue plasminogen activator (tPA) and matrix-degrading enzymes such as matrix metalloproteinase-9 (MMP-9) have been reported as potential circulating biomarkers for AAA diagnosis in case–control study [4]. Interestingly, SanGiorgi et al. [29] showed that MMP-3 and MMP-9 are effective monitors from 1 to 6 months after AAA surgery. However, these known circulating biomarkers associated with AAA could not be identified in the present study. The levels of these circulating biomarkers in AAA patients might remain high for a while after the surgery, and they might not change in the investigated periods (4 to 12 days) after the surgery compared with those in presurgery.

## Conclusions

Proteomic analyses of postsurgical sera compared with presurgical sera of patients with AAA and TAA were performed by using iTRAQ labeling followed by nanoLC-MALDI-TOF/TOF-MS/MS analysis. Among identified proteins with differential levels between postsurgical sera and presurgical sera, kallistatin in both AAA and TAA patients’ sera and α-2-macroglobulin in TAA patients’ sera might be candidates of serum biomarkers for aortic aneurysms. Our results provide valuable information on the underlining mechanisms of AAA and TAA and will contribute to the establishment of a method for early diagnosis of aortic aneurysm and to the development of pharmacological therapies.

## Methods

### Human serum samples

Blood samples were collected after approval from the Ethics Committee of Shimane University School of Medicine, Japan. The participants gave informed consent. Blood samples for quantitative proteome analyses were collected at presurgery and postsurgery from 7 AAA patients (5 males, 2 females; average age, 73.9 years) and 7 TAA patients (4 males, 3 females; average age, 73.1 years) who underwent aortic aneurysm resection during approximately 2 years in Shimane University Hospital. Presurgical and postsurgical blood samples were taken at an average of 7.1 days before and an average of 9.3 days after surgery, respectively. Atherosclerosis with calcification was macroscopically visible in most of the aneurysmal specimens from each patient. As normal control samples for quantitative proteome analyses, blood samples were collected from 4 healthy volunteers (2 males, 2 females; average age, 42.0 years) at two time points [first time point (T1) and second time point (T2)] with a 14-day interval. Age, gender, and time of sample collection of patients and control volunteers were shown in Additional file 1. Other presurgery and postsurgery blood samples were collected from patients with AAA (8 males, 4 females; average age, 79.4 years) and TAA (7 males, 3 females; average age, 67.6 years) who underwent aortic aneurysm resection during approximately 2 years in Shimane University Hospital for confirmation by Western blot analysis. Mixture of sera collected from 10 healthy volunteers (7 males, 3 females; average age, 42.0 years) was used as normal control samples for Western blot analysis. Age, gender, and time of sample collection of other patients and other control volunteers were shown in Additional file 2. Age matching of patients and volunteers was not fulfilled in the present study. Blood samples were centrifuged and the plasma layer was removed. Serum samples were stored at -80°C until use.

### Immunodepletion of abundant serum proteins

To remove the two most-abundant serum proteins [albumin and immunoglobulin (Ig)G], an immunodepletion column (Albumin & IgG depletion SpinTrap) from GE Healthcare (Buckinghamshire, UK) was used according to the manufacturer’s instructions. Briefly, the column was equilibrated with binding buffer (20 mM sodium phosphate, 0.15 M sodium chloride). After centrifugation, 50 μl of serum was diluted with an equivalent volume of binding buffer and then applied to the column and incubated for 5 min. After centrifugation for 30 s at 800 *g*, the eluate was collected. A further 100 μl of binding buffer was applied to the column and centrifuged. This elution step was performed again. The eluate was combined and then desalted, and its buffer was exchanged with 50 mM triethylammonium bicarbonate (TEAB) (Sigma, Tokyo, Japan) using spin concentrators (Corning, Tokyo, Japan). The protein concentration was determined using a bicinchoninic acid (BCA) Protein Assay Reagent (Thermo Fisher Scientific, Rockford, IL, USA).

### Denaturation, reduction, trypsin digestion, iTRAQ labeling, and strong cation exchange (SCX) chromatography

Sample preparation was performed accordingly to the manual supplied by AB Sciex (Foster, CA, USA) and our previous paper [30]. Briefly, equal amounts of immunodepleted presurgical and postsurgical serum samples were denatured by sodium dodecyl sulfate (SDS) and reduced by [tris-(2-carboxyethyl)phosphine (TCEP)]. Then cysteine alkylation was performed with methylmethanethiosulfonate (MMTS). Thereafter, each sample was digested by trypsin (AB Sciex). Each digest was labeled with a different iTRAQ tag by an iTRAQ Reagent Multiplex kit (AB Sciex). iTRAQ label 114 or 115 was used for labeling presurgical serum samples, and iTRAQ label 116 or 117 was used for postsurgical serum samples. Then the labeled presurgical and postsurgical samples were combined. This experiment was conducted for each patient. For the control samples collected at two time points, the former sample was labeled with iTRAQ label 114 or 115 and the latter sample was labeled with iTRAQ label 116 or 117. Then the labeled samples were combined. This experiment was conducted for each volunteer. After that, the combined samples were fractionated into six fractions by strong cation exchange (SCX) chromatography according to the instructions of the manufacturer (AB Sciex). Then each fraction was desalted by a Sep-Pac C_18_ cartridge according to the instructions of the manufacturer (Waters, Milford, MA, USA).

### NanoLC and MALDI-TOF/TOF MS/MS analysis

One fraction from SCX chromatography was fractionated to 171 spots with a DiNa nanoLC system (KYA Tech, Tokyo, Japan) and collected onto an Opti-TOF LC/MALDI 384 target plate (AB Sciex) using a Dina MaP fraction collector (KYA Tech) according to the instructions of the manufacturer (KYA Tech) and our previous paper [31]. Offline spotted peptide samples were analyzed on a 5800 MALDI-TOF/TOF MS/MS Analyzer with TOF/TOF Series software (version 4.0) (AB Sciex). MS spectra were acquired between *m*/*z* 800 and 4000 with positive ion mode. Parent ions of des-Arg1-bradykinin, angiotensin I, Glu1-fibrinopeptide B, adrenocorticotrophic hormone (ACTH) (clip 1–17), ACTH (clip 18–39) and ACTH (clip 7–38), diluted in a matrix [4 mg/ml α-cyano-4-hydroxycinnamic acid (CHCA), Wako, Osaka, Japan], were used for calibration. Monoisotopic precursor selection for MS/MS was carried out by automatic precursor selection with an interpretation method using the DynamicExit Algorithm (AB Sciex). MS/MS data obtained by the 5800 MALDI-TOF/TOF were analyzed by ProteinPilot™ software (version 3.0) with the Paragon protein database search algorithm (AB Sciex) [32]. Statistic method of iTRAQ analysis was according to ProteinPilot™ software (version 3.0). Each MS/MS spectrum was searched against the database constructed by AB Sciex (version 20081216, 20,489 entries). Based on the iTRAQ ratio of postsurgery to presurgery of each peptide, quatitative changes of each protein in presurgical and postsurgical sera were calculated. The comparison between the iTRAQ ratio of postsurgery to presurgery of patients and that of T2 to T1 of control volunteers was statistically performed by the unpaired *t*-test (two side test), with *p* < 0.05 being considered statistically significant.

### Bioinformatic analysis

Panther software (version 7.2) (http://www.pantherdb.org/) was used for protein classification analysis. The annotations of identified proteins were acquired from the Uniprot database (http://www.uniprot.org/) and appropriate literature.

### Western blot analysis

Crude sera for the analyses of α-2-macroglobulin, kallistatin, gelsolin and afamin or albumin- and IgG-depleted sera for the analyses of fibronectin and leucine-rich α-2-glycoprotein (LRG1) were electrophoresed through sodium dodecyl sulfate-polyacrylamide gel (SDS-PAGE), and then the proteins were transferred onto Hybond ECL nitrocellulose membranes (GE Healthcare Japan, Hino, Japan). The amount of crude sera (μl) and albumin- and IgG-depleted sera (μg) used for each analysis was as follows: α-2-macroglobulin (0.5 μl), kallistatin (2 μl), gelsolin (0.5 μl), afamin (2 μl), fibronectin (2.5 μg) and LRG1 (3.3 μg). Western blot analyses were performed as described in our previous paper [33]. The membranes were reacted with rabbit polyclonal anti-fibronectin antibody (Sigma-Aldrich, St. Louis, MO, USA), mouse monoclonal anti-LRG1 antibody (Abnova, Taipei, Taiwan), rabbit polyclonal anti-α-2-macroglobulin antibody (Abcam, Tokyo, Japan), goat polyclonal anti-kallistatin antibody (R & D Systems, Minneapolis, MN, USA), mouse monoclonal anti-gelsolin antibody (Sigma-Aldrich, St. Louis, MO, USA) or goat polyclonal anti-afamin antibody (GeneTex, Irvine, CA, USA). Then the proteins on the nitrocellulose membranes were reacted with anti-rabbit, anti-mouse or anti-goat Alex Flour 680-conjugated or IRDye 800-conjugated IgG (LI-COR, Lincoln, NE, USA), followed by visualization using the infrared imaging system Odyssey (LI-COR). The intensity of each band reacted with a corresponding antibody was measured for densitometric analyses of each protein level. Data from at least triplicate experiments were analyzed for statistical significance by the *t*-test (two side test). Its significance was set with *p* < 0.05. Results are expressed as means ± standard error (SE).

## Abbreviations

AAA: Abdominal aortic aneurysm; TAA: Thoracic aortic aneurysm; iTRAQ: Isobaric tag for relative and absolute quantitation; nanoLC: Nano liquid chromatography; MALDI: Matrix-assisted laser desorption ionization; TOF: Time of flight; MS: Mass spectrometry; MS/MS: Tandem mass spectrometry; LRG1: Leucine-rich α-2-glycoprotein; FN: Fibronectin; CBB: Coomassie brilliant blue; HDL: High-density lipoprotein; Apo: Apolipoprotein; Ig: Immunoglobulin; TEAB: Triethylammonium bicarbonate; BCA: Bicinchoninic acid; SCX: Strong cation exchange; SDS: Sodium dodecyl sulfate; TCEP: Tris-(2-carboxyethyl)phosphine; MMTS: Methylmethanethiosulfonate; ACTH: Adrenocorticotrophic hormone; CHCA: α-cyano-4-hydroxycinnamic acid; SDS-PAGE: Sodium dodecyl sulfate-polyacrylamide gel; SE: Standard error.

## Competing interests

The authors declare that they have no competing interests.

## Authors’ contributions

KS performed the sample preparation, peptide labeling and separation, MS analysis, raw data processing and analysis, bioinformatics, Western blot experiments, and statistic analysis. TM performed data analysis and bioinformatics. TO carried out sample collection and data interpretation. KM carried out the conception and design, data analysis and interpretation, and manuscript writing. All authors read and approved the final manuscript.

## Supplementary Material

Additional file 1 Sera used for iTRAQ labeling followed by nanoLC-MALDI-TOF/TOF-MS/MS analysis. Click here for file

Additional file 2 Sera of other patients used for Western blot analysis. Click here for file
