# Supplementary material for: Proteomic profiling for the identification of serum diagnostic biomarkers for abdominal and thoracic aortic aneurysms
Source: Proteome Sci. 2013 Jun 27;11:27. doi: 10.1186/1477-5956-11-27 (PMC3698092; doi:10.1186/1477-5956-11-27)
Supplement: Additional file 1 — Sera used for iTRAQ labeling followed by nanoLC-MALDI-TOF/TOF-MS/MS analysis. [file 1477-5956-11-27-S1.docx]

| Additional file 1 | | | | |  |
| --- | --- | --- | --- | --- | --- |
|  | | | | |  |
| *Sera used for iTRAQ labeling followed by nanoLC-MALDI-TOF/TOF-MS/MS analysis* | | | | | |
|  |  |  |  |  |  |
| Patients’ sera | | | | |  |
| AAA patient No. | gender | age | days before surgery | days after surgery | days between  the two sample collections |
| AAA1 | M | 58 | 3 | 4 | 7 |
| AAA2 | M | 83 | 14 | 7 | 21 |
| AAA3 | M | 77 | 4 | 8 | 12 |
| AAA4 | M | 63 | 7 | 8 | 15 |
| AAA5 | M | 77 | 9 | 11 | 20 |
| AAA6 | F | 84 | 5 | 12 | 17 |
| AAA7 | F | 75 | 11 | 12 | 23 |
|  |  |  |  |  |  |
| TAA patient No. | gender | age | days before surgery | days after surgery | days between  the two sample collections |
| TAA1 | M | 77 | 10 | 5 | 15 |
| TAA2 | F | 64 | 1 | 8 | 9 |
| TAA3 | F | 75 | 9 | 8 | 17 |
| TAA4 | M | 75 | 14 | 9 | 23 |
| TAA5 | M | 78 | 5 | 10 | 15 |
| TAA6 | M | 72 | 7 | 8 | 15 |
| TAA7 | F | 71 | 1 | 20 | 21 |
|  | | | | |  |
| Healthy control volunteers’ sera | | | | |  |
| Volunteer No. | gender | age | days between T1 and T2 | |  |
| C1 | M | 49 | 14 | |  |
| C2 | M | 45 | 14 | |  |
| C3 | F | 50 | 14 | |  |
| C4 | F | 24 | 14 | |  |
|  |  |  |  |  |  |
|  |  |  |  |  |  |
|  |  |  |  |  |  |
|  |  |  |  |  |  |
|  |  |  |  |  |  |
|  |  |  |  |  |  |
| Additional file 1, Satoh *et al.* | | | | | |
