# Supplementary material for: Proteomic profiling for the identification of serum diagnostic biomarkers for abdominal and thoracic aortic aneurysms
Source: Proteome Sci. 2013 Jun 27;11:27. doi: 10.1186/1477-5956-11-27 (PMC3698092; doi:10.1186/1477-5956-11-27)
Supplement: Additional file 2 — Sera of other patients used for Western blot analysis. [file 1477-5956-11-27-S2.docx]

| Additional file 2 | | | | |  |
| --- | --- | --- | --- | --- | --- |
|  | | | | |  |
| *Sera of other patients used for Western blot analysis* | | | | |  |
|  |  |  |  |  |  |
| Patients’ sera | | | | |  |
| AAA patient No. | gender | age | days before surgery | days after surgery | days between  the two sample collections |
| AAA8 | F | 79 | 6 | 6 | 12 |
| AAA9 | F | 86 | 7 | 11 | 18 |
| AAA10 | M | 82 | 3 | 6 | 9 |
| AAA11 | F | 84 | 3 | 10 | 13 |
| AAA12 | M | 61 | 4 | 11 | 15 |
| AAA13 | M | 89 | 4 | 6 | 10 |
| AAA14 | M | 77 | 2 | 7 | 9 |
| AAA15 | F | 80 | 4 | 10 | 14 |
| AAA16 | M | 89 | 5 | 4 | 9 |
| AAA17 | M | 89 | 1 | 75 | 76 |
| AAA18 | M | 72 | 2 | 10 | 12 |
| AAA19 | M | 65 | 9 | 10 | 19 |
|  | | | | |  |
| TAA patient No. | gender | age | days before surgery | days after surgery | days between  the two sample collections |
| TAA8 | F | 76 | 4 | 8 | 12 |
| TAA9 | F | 78 | 6 | 10 | 16 |
| TAA10 | M | 65 | 8 | 8 | 16 |
| TAA11 | M | 74 | 9 | 8 | 17 |
| TAA12 | M | 51 | 6 | 8 | 14 |
| TAA13 | M | 59 | 1 | 49 | 50 |
| TAA14 | F | 70 | 6 | 60 | 66 |
| TAA15 | M | 67 | 2 | 6 | 8 |
| TAA16 | M | 81 | 7 | 141 | 148 |
| TAA17 | M | 55 | 11 | 59 | 70 |
|  | | | | |  |
| Healthy control volunteers’ sera | | | | |  |
| Volunteer No. | gender | age |  | |  |
| C5 | F | 51 |  | |  |
| C6 | F | 37 |  | |  |
| C7 | M | 51 |  | |  |
| C8 | M | 52 |  | |  |
| C9 | F | 28 |  | |  |
| C10 | M | 55 |  | |  |
| C11 | M | 52 |  | |  |
| C12 | M | 48 |  | |  |
| C13 | M | 24 |  | |  |
| C14 | M | 22 |  | |  |
| Additional file 2, Satoh *et al.* | | | | | |
